# Split Azygos Vein: A Case Report

**DOI:** 10.7759/cureus.13362

**Published:** 2021-02-15

**Authors:** Stefan Lachkar, Joe Iwanaga, Emma Newton, Aaron S Dumont, R. Shane Tubbs

**Affiliations:** 1 Anatomy, Seattle Chirdren's, Seattle, USA; 2 Neurosurgery, Tulane University School of Medicine, New Orleans, USA

**Keywords:** inferior vena cava, embryology, azygos vein, variation, anatomy, cadaver

## Abstract

The azygos venous system, which comprises the azygos, hemiazygos, and accessory hemiazygos veins, assists in blood drainage into the superior vena cava (SVC) from the thoracic cage and portions of the posterior mediastinum. Routine dissection of a fresh-frozen cadaveric specimen revealed a split azygos vein. The azygos vein branched off the inferior vena cava (IVC) at the level of the second lumbar vertebra as a single trunk and then split into two tributaries after forming a venous plexus. The right side of this system drained into the SVC and, inferiorly, the collective system drained into the IVC. Variant forms in the venous system, especially the vena cavae, are prone to dilation and tortuosity, leading to an increased likelihood of injury. Knowledge of the anatomical variations of the azygos vein is important for surgeons who use an anterior approach to the spine for diverse procedures.

## Introduction

The inferior vena cava (IVC) is the largest vein in the human body. Its principal function is to return venous blood from the abdomen and lower extremities to the right atrium of the heart [1]. Developmental patterning of the IVC consists of three paired embryonic veins: subcardinal, supracardinal, and postcardinal. These veins go through recurrent phases of regression and progression, forming various anastomotic connections and ultimately giving rise to the final, mature vena cava system [2].

Anatomically, the azygos vein originates from the connection of the right subcostal vein and right ascending lumbar veins at the T12 vertebral level [3]. It enters the thorax through either the aortic hiatus or posterior to the right crus of the diaphragm, ascending along the anterolateral surface of the right thoracic vertebra [3]. Ultimately, it courses superiorly, uniting with the superior vena cava (SVC) at the level of the fourth thoracic vertebra. The main and most numerous tributaries draining into the azygos vein are the posterior intercostal veins. Superiorly, the azygos drains into the SVC. In view of the complexity of the various embryological stages of the IVC, numerous variant conditions can be attributed to defects in the three paired embryonic veins. In this report, we discuss a unique case of a variant azygos vein.

## Case presentation

**Figure 1:**
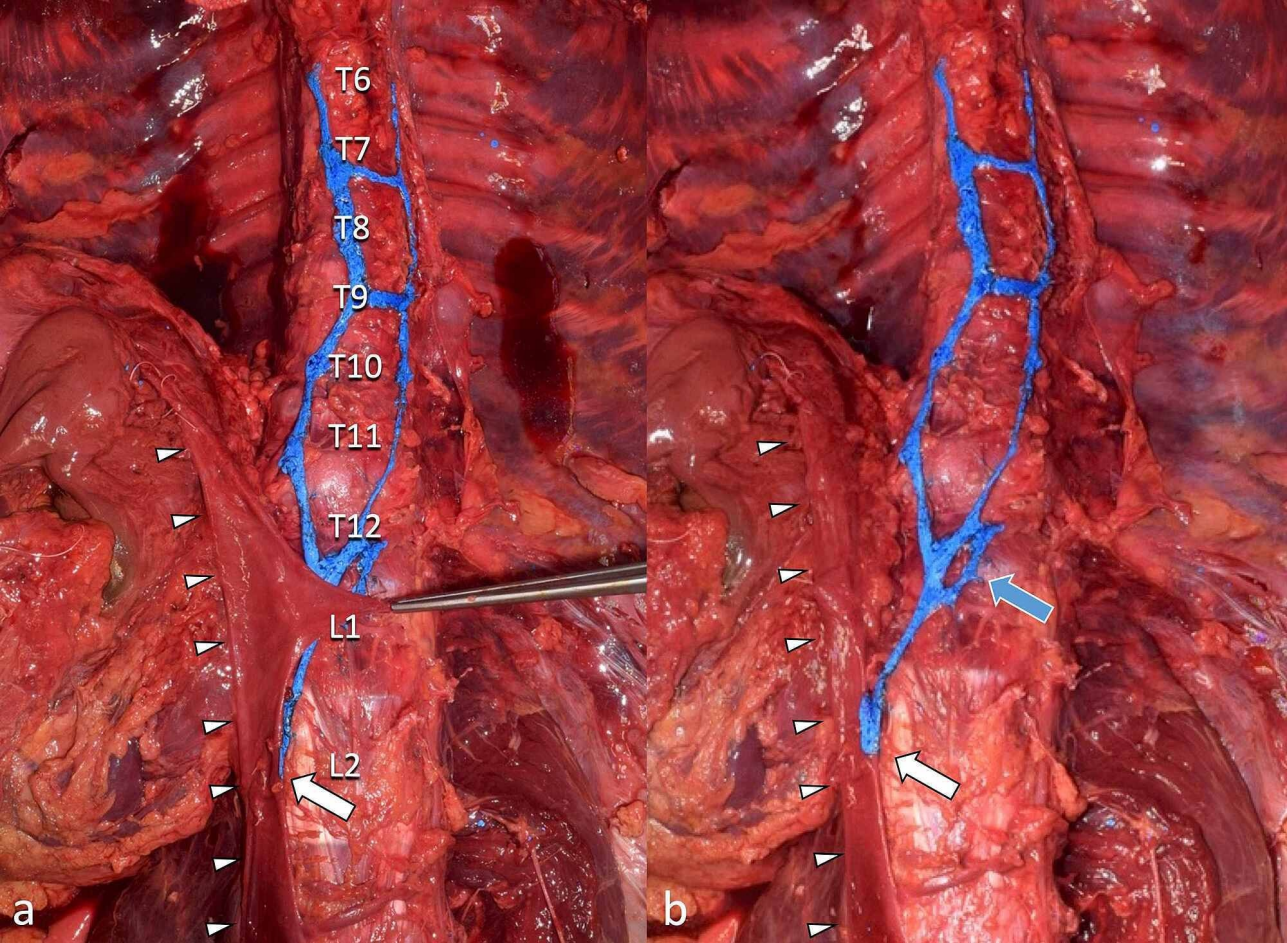
Split azygos vein arising from the IVC (arrowheads) at the level of L2 (white arrows) a: left renal vein retracted to the left side; b: left renal vein reflected to the right side Note the azygos vein giving rise to venous plexus (blue arrow) before splitting IVC: inferior vena cava

A split azygos vein was found during routine dissection of the fresh-frozen cadaveric specimen of a Caucasian male who had been 46 years old at the time of death. The azygos vein branched off the IVC at the level of the second lumbar vertebra and ascended medially, forming a small venous plexus and splitting into two tributaries between the T12 and L1 levels of the vertebral column. The right tributary drained into the SVC while the left drained from the intercostal veins (Figure 1).

**Figure 2:**
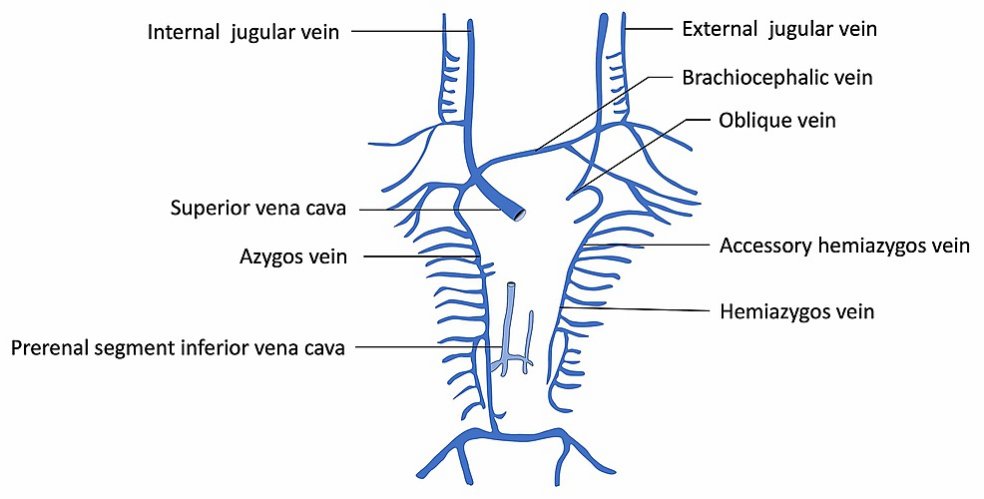
Schematic drawing of the normal development of the venous system

Neither a hemiazygos nor an accessory hemiazygos vein was found on the left side. The posterior intercostal veins (not shown) drained into the left and right sides of the variant system shown in Figure 2. The right-side venous chain drained into the SVC. The left chain was contiguous with the left third posterior intercostal vein. No other anatomical variations in the vena cava system or surrounding structures were noted during dissection.

## Discussion

Embryological development 

Embryological development of the IVC begins between four and eight weeks after conception and entails the recurrent regression and appearance of paired embryonic veins: subcardinal, posterior cardinal, and supracardinal [1,4]. During embryonic development, these three paired veins go through continual regression and anastomosis until the mature IVC is formed. The first major embryonic event is the regression of the paired umbilical veins, which drain blood from the placenta, and the left vitelline vein, which drains blood from the yolk sac [4]. This gives rise to the hepatic segment of the IVC, formed from the persistence of the right vitelline vein. Around the 35th day of gestation, the appearance of the subcardinal veins and the associated intersubcardinal anastomosis progressively take over the task of draining venous blood from the lower half of the body, previously done by the posterior cardinal veins [5]. The connection between the right subcardinal vein and the hepatic segment of the IVC emerges, forming the suprarenal and hepatic portions of the IVC, respectively. Around the seventh week of gestation, the supracardinal veins emerge and take over venous drainage of the lower half of the body from the subcardinal veins [5]. The right supracardinal vein persists, and the regression of the left supracardinal vein gives rise to the infrarenal segment of the IVC. The cranial portion of the right supracardinal also gives rise to the azygos vein. The right subsupracardinal anastomosis develops into the renal segment of the IVC [1]. The final mature IVC develops by the eighth week of gestation and comprises four segments: hepatic, suprarenal, renal, and infrarenal (Figure 2) [1].

Anatomical variations

Because the embryological development of the IVC is multifaceted, there can be embryological derailments at any stage. Variants of the IVC can be classified according to the embryonic vein precursors [5]. Mathews et al. have described assorted variants derived from the posterior cardinal, subcardinal, and supracardinal veins, and the renal segment of the IVC. Persistence of the right posterior cardinal vein leads to a retrocaval ureter, moderately circumventing the IVC [5]. Developmental abnormalities of the subcardinal veins can lead to interruption of the IVC, resulting in blood being returned to the heart via the azygos/hemiazygos continuation. The supracardinal vein develops into the infrarenal segment of the IVC. Persistence of the left supracardinal vein together with regression of the right results in the formation of a left-sided IVC. Doubled vena cava variations occur when the subcardinal vessels persist, either partially or completely. A right-sided IVC is commonly deemed dominant, while a left-sided IVC anastomoses with the right side through a series of veins, effectively establishing a venous collar that surrounds the aorta.

Azygos vein variants can be classified into three types - type 1: two vertical veins with no connection points; type 2: an anastomotic connection dorsal to the aorta between the azygos and hemiazygos systems; and type 3: a single azygos vein located at the midline [6]. Studies by Seib (1934), Anson and McVay (1984), Kutoglu et al. (2012), and Dahran and Soames (2016) indicate that type 2 is the most common azygos venous system variant while type 1 is the rarest [6-9].

Nayak and Soumya [10] have described a reversed azygos system with the azygos shifted to the left side and draining into the brachiocephalic vein and the accessory and hemiazygos system shifted to the right side of the body. Badagabettu et al. [11] have reported a hemiazygos vein with multiple linkages to the azygos vein across the midline. Neither of the cases reported by these authors or others was similar to our case, where two more or less vertical venous chains converged into a single vein inferiorly to drain into the IVC and, just prior to this, formed a circular venous plexus.

A venous plexus and split azygos vein were observed in the present case. This variant could have resulted from abnormal regression and/or persistence of the subcardinal and supracardinal veins, and intersubcardinal anastomosis during the fourth to eighth weeks of gestation. This variant is seemingly uncommon due to the plexiform nature of its thoracic parts and the drainage into the IVC of this collective system. 

Clinical significance

Anatomical variations in the venous system, especially the vena cava, are of utmost importance to surgeons and interventionalists. For example, there can be thromboembolic complications in the variant vena cava, increasing the risk of pulmonary embolism [5]. Potential problems arise when clinicians reviewing medical images misdiagnose a doubled IVC as mediastinal lymphadenopathy or a para-aortic mass, leading to the unnecessary risk associated with a biopsy [12]. Since minimally invasive vascular approaches, especially via the venous system, are on the rise currently, anatomical venous variants are now even more important to the clinician than ever before. Moreover, surgically, such an anatomical variation, as demonstrated in the present case, could result in catastrophic hemorrhage if not recognized either with preoperative imaging or during surgical approaches. Therefore, a thorough knowledge of their potential variations can improve patient care [13-15].

Acknowledgment

The authors sincerely thank those who donated their bodies to science so that anatomical research could be performed. Results from such research can potentially enhance mankind’s overall knowledge and thus improve patient care. Therefore, these donors and their families deserve our highest gratitude [16].

## Conclusions

The IVC develops embryologically through a series of multifaceted events involving the recurrent regression and formation of three paired embryonic veins while the final mature venous system is forming. Anatomical variations involving the IVC and the azygos venous system can lead to potential complications for surgeons and clinicians alike.
